# Gender heterophily and equality: a contribution to gender equality in the Chilean scientific sector

**DOI:** 10.3389/fpsyg.2023.1032291

**Published:** 2023-09-05

**Authors:** Juan Felipe Espinosa-Cristia, Alejandro Vega-Muñoz, Luis Manuel Cerda-Suarez, Luis Leyton-Johns

**Affiliations:** ^1^Departamento de Ingeniería Comercial, Universidad Técnica Federico Santa María, Valparaíso, Chile; ^2^Instituto de Investigación y Postgrado, Facultad de Ciencias de la Salud, Universidad Central de Chile, Santiago, Chile; ^3^Public Policy Observatory, Universidad Autónoma de Chile, Santiago, Chile; ^4^Facultad de Economía y Negocios, Universidad Andres Bello, Santiago, Chile

**Keywords:** homophily, heterophily, women and science, social sustainability, gender equality, SDG 5

## Abstract

Women’s insertion or consolidation in science has been thoroughly researched. Such discussion could be particularly relevant concerning sustainable development goal five (SDG 5) on Gender Equality advancement. However, the debate is focused on women percentages inserted into scientific labor, leaving the issue of symbolic experience for women in research unaddressed and with little empirical support. The data come from a survey developed under a FONDECYT project, which studied knowledge production in Chile. Researchers obtained contacts through invitations on social networks such as Twitter, Facebook, and LinkedIn and databases containing scientists’ emails working in Chile. The non-probabilistic sample collected 583 cases, with participants residing in 15 of the 16 country regions. As a result, this document presents the findings of a study on symbolic experience using an instrument to determine whether there are any homophily patterns. It aims to determine if scientists tend to cite others as referents only when they encounter a situation like their own. The findings reveal a clear way scientists estimate the effect of others in terms of their gender homophily. This intervening factor could be crucial in reproducing the disparities and asymmetries that characterize Chile’s scientific field.

## 1. Introduction

### 1.1. Women and science in Chile

In general terms, gender inequities accumulate across the employee lifespan and at multiple levels, and gender differences in the workplace arise from inequitable treatment and outcomes (Son Hing et al., 2023). In Sustainable Development Goal 5-Gender Equality-masculine and gender-set-apart areas, such as the extractive industry, show crucial constraints for women’s sustainable leadership chances and career opportunities (Franco et al., 2020). The literature shows that society needs to resolve those constraints in allegedly more diverse fields like academia and research-based activities. Indeed, social scientists discuss the insertion and consolidation problem of women in science widely (Tiedemann, 2002; UNESCO, 2017a,b, 2018; Thébaud and Charles, 2018; McGuire et al., 2020; Galvan et al., 2021). According to UNESCO (2017a,b).

Furthermore, women’s participation in higher education has advanced significantly in the last decade, especially in terms of access; women continue to be a numerical minority within the various scientific contexts, making up 28.8% of the total researchers worldwide and 45.4% of the Latin American level. One of the most emblematic cases of gender segregation in research is the case of disciplines in Science, Technology, Engineering, and Mathematics (STEM). In these disciplines, women not only face significant glass ceilings that make it difficult for them to move up the academic ladder (ONU Mujeres, 2019), but they also face horizontal segregation due to low female representation in these scientific disciplines, where only 35% of the total enrollment in STEM undergraduate programs define themselves as women (UNESCO, 2018). The persistence of vertical (or glass ceiling) and horizontal segregation, both at the educational and occupational level, contribute to reproducing the gendered stereotypes present in science, strengthening the idea that men are better in hard science or engineering discipline fields and women are innately talented with soft skills, expression, and caring for others (Tiedemann, 2002; McGuire et al., 2020; Galvan et al., 2021; Filandri et al., 2023). This aspect is relevant because experts show that women have qualities that can be vital for success in STEM disciplines. Shauman and Huynh (2023) point out that interventions designed to reduce biases should be rooted in well-supported theories about prejudice and bias reduction. In this line, the prejudice habit theory conceptualizes bias as a mental habit. It lays out the steps needed to break this biased habit. Researchers, for example, use status theory to frame disparities in postdoctoral hiring, articulating how status drives the cultural construction of distinct social groups, becoming a direct cause of inequalities between researchers (Bellotti et al., 2022).

In the Chilean case, while women occupy 51.3% of undergraduate enrollment in scientific work, the percentage of women is only 22% (Dinamarca, 2020). These data indicate that as one of the advances in the academic career, the gender gap increases. As the Chilean National Commission for Scientific and Technological Research pointed out, the number of women applicants to research funds and grants reached 41% of the total, while men reached 59%. However, the gender gap in projects awarded since 2015 has steadily decreased for the publication year of this report. The percentage of projects awarded a project or grants led by women was 39.9%, with an award rate of 29.9% for women (Female award rate = (No. of awarded projects led by women in year t/ no. of eligible projects led by women in year t)*100) (CONICYT, 2018). In line with this, of the total number of researchers, only 28% are women, and only 16% lead high-performance scientific teams (Comisión Nacional de Investigación Científica y Tecnológica (CONICYT), 2017). Comunidad Mujer (2017) states that female underrepresentation is more evident in STEM areas and more markedly so in technological areas. This underrepresentation occurs both at the enrollment level in related careers and in participation in the academic bodies of universities. The cited underrepresentation would place Chile among the cases with the most significant gender gap in this area in countries that are part of the OECD.

The debate seems to focus nationally and internationally on the percentage of women’s insertion in scientific work. To cite an essential and actual example, this gender imbalance regarding the number and composition of leaders females’ and males’ authorship got worse during the COVID−19 pandemic (Liu et al., 2022). However, the symbolic experience problem of women in science has had less empirical treatment. These data show a scenario of unequal representation in terms of numbers and indicate the symbolically adverse labor context women scientists must endure daily. As Kiss et al. (2007) point out, female scientists work in alien and adverse spaces. As indicated by several studies (Acker, 1990; Martínez Labrín, 2012; O’Connor et al., 2015; Arêas et al., 2023), scientists and general population discourses about science are androcentric as everyday relationships in the contemporary academic culture. These androcentric discourses and relations tend to (re)produce the preponderant gender stereotypes in society in at least two ways.

On the one hand, they reproduce the classical sexual labor division in which women should be concerned with care and administration tasks, and men engage in productive work (Bird and Codding, 2015). General discourses on woman’s science work maintain the categorizations of cognitive inferiority attributed to women compared to men (Hochschild, 2003; Ahmed and Olivares-Mansuy, 2014). As Acker (1990) claims, academics built an “ideal scholar” figure as a highly productive, accessible, and independent male body without any responsibility for caregiving based on gender stereotypes. Male and female scientists must execute this ideal if they wish to be recognized as successful scholars within university spaces (Davies and Gannon, 2006), a situation that threatens the advancement of women in scientific careers.

Any sustainability and SDG 5 analysis are inherently interdisciplinary (Bellotti et al., 2022). Furthermore, the literature points out that several theories, such as feminist caring theory, social role theory, gender identity theory, resource dependence theory, and upper echelons theory, explain women’s leadership’s influence on sustainability organizational behavior. Several authors argue that a single theory cannot fully explain the effect of women’s leadership on sustainability proactivity and suggest combining several theoretical perspectives (Son Hing et al., 2023). For example, according to social role theory, scientists believe that sexes constitute gender roles, fostering fundamental behavior differences through various mediating processes. Similarly, upper echelons theory points out that the demographic traits and experiences in leadership positions in institutions shape their values and management styles, which in turn influence their decisions. In addition, according to dependence resource theory, women bring distinctive resources and capabilities to organizations in terms of knowledge, expertise, and skills (Cerdá et al., 2023).

In this context, and to enlighten one of the less-studied dimensions, the present study looks to characterize the network of male and female scientists and how they relate to each other by openly mentioning their referents in their scientific careers. While public discussion about gender equal equity has been in vogue in recent years in the field of research and science (Burton, 1857), there is a dimension of women’s work in science that has been less visited, which is the possibility for women to find referents in other women scientists. This study aims to observe how women see themselves represented by other women in the scientific field in Chile. To do so, we based on the concept of homophily, a widely used term by social scientists. As seen in the definition proposed by Lazarsfeld and Merton (1954), “many social networks show what has been called homophily… the tendency of people to choose to interact with similar others” (McPherson et al., 2001, p. 37). Burton (1857) develops the concept of homophily in his famous phrase “birds of a feather go together” to analyze this social behavior. Homophily is essential in social network logic, as highly segregated networks are affected by information access terms about jobs and different information sources and behavior. Consequently, studying the phenomenon of homophily in science may shed light on gender segregation in the scientific field, which may have essential implications in revealing how male and female scientists access work and how they relate to each other.

This paper aims to contribute to the growing discussion on women in science, which, as per the new public policy enacted by the Chilean Ministry of Science, Knowledge, Technology, and Innovation (Biblioteca Nacional del Congreso, 2020), specifically from its new advisory council on gender and inclusion, has gained relevance in this country. In the same vein, the paper looks to contribute to social sustainability goals, being able to be used as an argumentative basis for creating more equalitarian opportunities for men and women (SDG 5).

The article begins by reviewing the literature in the field of social networks and the concepts of homophily and its application in the study of symbolic relational aspects for the field of science and the phenomenon of gender in science. The authors then describe the methodology, emphasizing gender homophily calculations with the collected data. Subsequently, the article presents results by gender. Finally, the authors discuss the results and an understanding of what the Chilean case can contribute to the discussion on gender homophily in the scientific field. In the end, the article presents some conclusions of the study.

### 1.2. Networks, homophily, and science activities

The concept of homophily has an essential application in the social network analysis field. Social scientists identify several dimensions to define homophily. In the scientific networks case with emphasis on gender, social scientists divide the study of homophily into at least three dimensions: (1) Data obtention, (2) network modeling, and (3) substantive aspects (e.g., scientific collaboration or labor hiring). These dimensions enable, on the one hand, responding to the debate on the women’s role and participation in the production of science, and on the other, posing new challenges in understanding this phenomenon in Chile.

As with any study from a social network perspective, the focus or interest needs to be specified, i.e., if it is a socio-centric or egocentric approach (Perry et al., 2018). The former refers to how individuals are integrated into each context, describing all the links for the structure. Instead, egocentric approaches focus on specific people or ego contacts (McCarty et al., 2019). Such specification has a direct impact, at least on data collection and network modeling methods.

Concerning certain substantive aspects, social scientists associate collaboration in science with concepts of network analysis, such as homophily, transitivity, and preferential connection. The literature that has studied the role of homophily in the process of collaboration allows us to understand the various scenarios and variables that show the relationship between homophily, heterophylly, and collaboration (Boschini and Sjögren, 2007; Sie et al., 2012; Freeman and Huang, 2014; Zhang et al., 2017; Holman and Morandin, 2019). Homophily would facilitate communication and reduce certain costs, which benefits scientific collaboration. While measuring homophily depends on the traits of the actors under consideration, a favorable trend toward homophily in scientific collaboration has been highlighted in the literature when assessed by gender. In other words, the scientific context evidence from these articles would indicate a tendency to collaborate with others of the same gender when working on an article to be published.

It is worth noting that scholars studied gender homophily in a broader employment context. For example, the relationship between homophily and job hires has been studied by Edo et al. (2019), concluding that having similarities with recruiters, for example, when applicants and recruiters are all males, they tend to have better opportunities. Similarly, according to that study, people showcase favoritism when women are recruiters and are more likely to choose other women for jobs. In France, recruiters are biased on gender, class, ethnicity, and local stereotypes. Homophily found in recruitment is an example of social networking models that describe stereotype-reproducing practices that impact how people find career opportunities.

Furthermore, the homophily found in recruitment processes draws attention to the relationship between gender and society in the configuration of the various fields (Brashears, 2008). For example, in the context of business innovation, literature reports that female empowerment leads to women showing superior, innovative activity to men. In fact, in recent decades, women have been found to have advanced in the dynamics of fostering gender equality through innovation in terms of less fear of failure and improving their standard of living, among others. Their empowerment is related to society and involves seizing opportunities by drawing on acquired knowledge, culture, and customs, which they combine with creativity to give rise to innovation (Cerdá et al., 2023).

Thus, Brashears comments that stereotype reproductive practices are related to gaining social capital and macro structures within the operations of the studied space. In this sense, the associations we form with others are determined by our society, not by sex (Brashears, 2008). In this line, we can observe the relevance of the model we have defined of social relations based on previous criteria, which come from social capital. Therefore, scholars understand gender as a gravitate variable due to the structure of social capital associated with gender in each culture or society. With this, social scientists understood that “researchers should attempt to identify the concrete sources of the differences in male and female social distances and homophily for non-kin relations. These differences either reflect different structural constraints or suggest that, for some reason, the same constraints affect males and females in different ways” (Edo et al., 2019, p. 413). Thus, part of this work seeks to contribute to understanding the relationship structure between associativity and gender.

Homophily, then, has been understood as a social mechanism explaining certain behaviors that it reproduces. Observing egocentric networks will allow us to understand the tendency towards association among agents with similar attributes (men who recommend or acknowledge men as points of reference). In the case of gender, the study of egocentric networks is fascinating. Even though people relate to agents of the other gender in research fields, structures of social relations, in this case, still favor men to the detriment of women.

## 2. Methodology

### 2.1. Materials and methods

As stated in the previous section, social scientists approach the homophily analysis from a socio-centric or egocentric perspective. The socio-centric perspective studies scientific networks and has been based on the collection of bibliographic references (Bravo-Hermsdorff et al., 2019), co-authorships (Boschini and Sjögren, 2007; Sie et al., 2012), and finally, studies of research trajectories of decision makers and their respective research fields (Collins and Steffen-Fluhr, 2019). In this case, this article approaches the homophily study from the egocentric perspective; thus, the results will always depend on how the ego network is bounded and characterized (Ofem et al., 2013; Crossley et al., 2015; Perry et al., 2018; Levinson et al., 2022). This article used a simple question with no name-generator describing additional information from the alter.

For example, (Laniado et al., 2016) highlight that dyadic and triadic structures occur and that identifying homophily and heterophily is a normative target concept that depends on the context (for example, in discussing spaces of incoming inequality). Kovanen et al. (2013) state a prevalence of all-female triangle motifs reported for phone calls in a large dataset of mobile phone records and show the existence of temporal homophily in those structures. For our case, as we further developed in this section, we built our modeling and designing an instrument that looks to account for the interaction and representation of women in scientific work and to identify whether there are patterns of homophily (McPherson et al., 2001) among male and female scientists working in laboratories in Chile. In other words, this instrument seeks to reveal whether people working in science tend to mention others as their referents only if they had some condition like their own.

In terms of the research techniques, the study draws from a survey developed under a Chilean state funded project (FONDECYT), which studied the production of scientific knowledge in Chilean Laboratories. The non-probabilistic sample collected 583 valid cases, with participants residing in 15 of the 16 regions of the country. Table 1 shows the distribution concerning areas of knowledge in the sample. The purpose of the survey was to collect information about the experience in the scientific field, the organization of work, working conditions, and the motivation and trajectory of individuals within the organization.

**Table 1:** Distribution with respect to areas of knowledge in the sample.

Areas of Knowledge	Men	Women	Total
Agricultural Sciences	8.5%	7.7%	8.2%
Natural Sciences	43.4%	37.0%	40.9%
Social Sciences	5.0%	6.1%	5.4%
Arts and Humanities	1.4%	2.8%	2.0%
Technology and Engineering	15.7%	10.0%	13.4%
Medicine and Health Sciences	21.0%	32.0%	25.4%
Non Specified Field	5.0%	4.4%	4.8%
Total (n=)	281	302	583

Researchers implemented the questionnaire using the website Typeform. Typeform allowed the pre-analysis of the responses “online.” The instruments contain 53 questions about different knowledge production aspects related to researchers. There are questions about the demographics of researchers (In which region of the country is your laboratory located?), about gender: Which gender do you identify with? and others about their perception of the Chilean Science Policy (How much do you agree with the following sentence? “The institutionalization of knowledge in the country seems to be adequate”). All in all, one of the questions included in the questionnaire allowed for an egocentric network approach. This question (number 49) was: “Name the three people in Chile who have had the most influence on your career.” This information provided up to three alters who influenced the ego’s career (i.e., three people other than the ego). Although the authors did not explicitly ask for additional information on each of these people, we could identify the gender characteristic of each of them by their names or roles (for example, father or mother). The authors could not identify the gender on 12 occasions. In those cases, the study assigned the condition of the “unidentifiable” gender.

Regarding the practicalities of the non-probabilistic sample, the authors obtained contacts through invitations on social networks such as Twitter, Facebook, and LinkedIn and databases containing emails of scientists working in Chile. The authors also contacted research organizations and large research Chilean Centers using their network and networks built upon their extensive ethnographic previous fieldwork of the research project. Then, using emailing-based distribution, the survey was sent to the complete list of email contacts those organizations facilitated to the authors. Furthermore, one of the assistants of the research project web scrap some researchers’ emails to increase the database contacts for the survey target.

In terms of the methodology and to better understand the homophily analysis presented in this research, some theoretical aspects should be considered. Following (McPherson et al., 2001), we can distinguish two types of homophily. On the one hand, there is baseline homophily when the demography of possible potential links creates these patterns. On the other hand, inbreeding homophily occurs when such patterns appear beyond demography. Inbreeding homophily is close related to social similarity. The latter would typically be motivated by preferences and tastes (Bargsted et al., 2020).

The analysis conducted in this study represents an ascription of part of the first level defined above. In other stages of life, mainly in the early stages of socialization, patterns of homophily between men and women are often defined by preferences rather than bonding potential. McPherson et al. (2001) show a more detailed review of inbreeding homophily. However, on this occasion, the analyzes are oriented towards a specific work activity, where the number represented by men and women is not balanced. Homophily, in this case, should be associated only with a tendency to mention those with identical characteristics in terms of gender as influential people, considering that men and women do not represent similar proportions in participation in scientific work. Along this line, and echoing the findings in the literature, it would be expected that the minority group, in this case, women, would have networks that are more heterophilic than the majority group, as commented by Collins and Steffen-Fluhr (2019).

Therefore, to obtain the variables that would allow the analysis described up to this point, the question “Name three people in Chile who have been most influential in your career” was considered. The study named the first alter 1, the second to alter 2, and the third to alter 3. In this article, the authors manually input each alter’s gender based on name and surname mentions. In other cases, such as generic mentions, gender was input depending on whether it was possible. In cases where it was not, the authors assigned the category: unidentifiable.

Referents in one to three cases to a given gender by both male and female researchers allow to calculate a weighted average number of referents by gender from a given gender, thus establishing referents intensities in a two-by-two transition matrix, which, being irreducible, converge to a stationary state indicating how this set of current intra-gender and inter-gender relationships tends to lean towards referents of one gender or the other (Abelson, 1967; Proskurnikov and Tempo, 2017).

On the other hand, the authors estimated four variables. The first, called E, is the number of ties or links with the same gender characteristics as the ego. The second, called I, is the number of ties that differ from the ego in gender. The third is the difference or subtraction between E and I. Finally, the fourth is the total number of ego ties or the sum of E and I.

With the last four variables presented here, the authors estimated homophily by the ‘EI’ index (Krackhardt and Stern, 1988). Such an index has been recently used in studies about research communities (see Leifeld, 2018; Hopkins et al., 2019; Locatelli et al., 2021; Hangül et al., 2022). Furthermore, social scientists have used the EI index to study research communities and their relations with gender (Yamamoto et al., 2018).

This estimation is the division of the difference between ties with equal characteristics and ties with characteristics different from the ego. That is, the third variable, which is the difference or subtraction between E and I regarding the total sum of links of the ego—or the fourth variable, which is the sum between E and I. The following equation illustrates the index:


$$EI=\frac{E-I}{E+I}$$

For example, If the ego -the person who completed the survey- is a woman, she mentioned one man and two women as the people in Chile have worked and most influenced her career.

This result, which has a value of 0.33, indicates that it is a mixed network, as it is not an integer, where there is a favorable tendency toward homophily because its value is positive and different from zero. The EI index can take values ranging from −1 or total heterophily, in which all the members of the network are different from the ego, and 1 of total homophily, where all the members of the network are equal to the ego in the considered characteristic.

### 2.2. Descriptive analysis of the general structure

First, of the total number of respondents, how many mentioned a person in Chile who has been influential in their career? Table 1 summarizes this information.

Of the surveyed people, 19% did not mention any person in Chile who had been influential in their career. On the contrary, 81% of the respondents mentioned at least one person, 20% mentioned one referent, 10% mentioned two referents, and 51% mentioned three referents in Chile for their scientific career.

## 3. Results

### 3.1. Descriptive analysis

In results terms, the total number of mentions made is 1,130, with 925 referents identified without repetitions. Table 2 shows the number of mentions this study categorized into female, male, and unidentifiable.

**Table 2:** Percentage of the number of mentions of the full sample.

Mentions	Frequency	Percentage
0	111	19%
1	114	20%
2	58	10%
3	300	51%
> 1	472	81%
Total	583	100%

For the analysis presented below, considering a total of 472, all the networks that have only one alter and are unidentifiable in the gender category—8 networks—are eliminated, in addition to the two networks in which all the alters were unidentifiable in gender. Thus, the total number of egocentric networks to be analyzed is 462.

**Table 3:** Gender composition of the total number of mentions.

Gender	Frequency	Percentage
Female	268	24%
Male	839	74%
*Unidentifiable*	23	2%
Total	1,130	100%

In 17 networks, there was one person whose gender could not be determined, and in 2 networks, all the alters fell in the category of unidentifiable in terms of gender. Of the total mentions, 106 networks are sized 1, 59 networks of size 2, and 298 networks are of size 3, i.e., 23% of networks are size 1 (or grade 1), 13% are size 2 (or grade 2), and 64% of the scientific influence networks size 3 (or grade 3).

Within these structures, we ask the question: What is the proportion of men and women who are considered influential in Chile for scientific careers? In Table 3, this study presents detailed information about the network size at a general level. Finally, the authors eliminated problematic cases for the analysis of homophily.

**Table 4:** Distribution of mentions to female, male, and non-identifiable referents.

Number of mentions	Mentions to female referents	Mentions to male referents
0	256 (55%)	37 (8%)
1	152 (33%)	151 (33%)
2	46 (10%)	134 (29%)
3	8 (2%)	140 (30%)
Total	462 (100%)	462 (100%)

The results in Table 4 indicate that this tendency towards homophily is only valid for those who identify with the male gender and that the average value of the ‘EI’ index of the sample of 462 egocentric networks is attenuated by the egocentric networks of women. In the case of female scientists, the tendency toward heterophilia; when mentioning people in Chile who have influenced their careers, these women tend to mention both their male and female peers. However, it is noteworthy that only men represent 43% of women’s networks of influence. In 23% of the networks where the ego is female, two-thirds corresponded to men. In 10% of the networks, there is a balance between women and men. The cases of predominantly women in their networks are only 24, 14% of which two-thirds are of the same gender, and 10% are all women.

Regarding mentions of the female gender, in 55% of the networks, subjects did not mention women as referents. Of 33%, subjects mentioned just one woman; furthermore, 10% mentioned two women as referents, and 2% of the subjects mentioned three.

On the other hand, male referents were not present in 8% of the networks; in 33% of the networks, there is only one person of the male gender. In 29%, subjects mentioned two males as referents; in 30%, three were mentioned as influences. Finally, in the indeterminate mentions, in nine networks there is one person of indeterminate gender (2% of the network). All these cases in the EI formula by Krackhardt and Stern (1988), were considered as different from the ego in gender.

### 3.2. Gender homophily analysis

As mentioned in the data and methods section, the authors estimated the Krackhardt EI index for each of the 462 egocentric networks. Table 5 shows the distribution of the values.

**Table 5:** Krackhardt and Stern EI index of scientific networks of influence.

Value	Frequency	Percentage
−1 (heterophily)	94	20%
−0.33	59	13%
0	28	6%
+0.33	88	19%
+1 (homophily)	193	42%

In 20% of the network, the people mentioned are different from the ego regarding gender. In 13% of the network, two-thirds of the referents differ from the ego (One could also say that “one-third have similar characteristics,” but since heterophily is the tendency to share something with different others, people usually mention the difference); in 6% of the networks, there is a balance in the distribution, which can only occur when there are two referents, as the limit of alters is three. On the other hand, in 19% of the networks, two-thirds of the mentioned alters the same gender characteristics as the ego. Additionally, in 42% of the egocentric networks, all alter are of the same gender. There is a tendency towards homophily when mentioning people in Chile who have been influential in scientific careers. However, is this tendency common to both men and women or are there differences by gender? We can look at the information shown in Table 6.

**Table 6:** Distribution of mentions to female, male referents from each gender.

Criteria	# Mentions	Male (*n = 281*)	Female (*n = 181*)	Total
Total Mentions	1	66 (23%)	40 (22%)	106 (23%)
	2	30 (11%)	28 (16%)	58 (13%)
	3	185 (66%)	113 (62%)	298 (65%)
Total weighted mentions		2.42	2.40	
Mentions to female referents	0	178 (63%)	78 (43%)	256 (55%)
	1	82 (29%)	70 (39%)	152 (33%)
	2	19 (7%)	27 (15%)	46 (10%)
	3	2 (1%)	6 (3%)	8 (2%)
Weighted mentions to female referents		0.45	0.78	
Mentions to male referents	0	17 (6%)	20 (11%)	37 (8%)
	1	80 (28%)	71 (39%)	151 (33%)
	2	83 (30%)	51 (28%)	134 (29%)
	3	101 (36%)	39 (22%)	140 (30%)
Weighted mentions to male referents		1.95	1.60	

First, regarding the mentions in general, it is noted that men and women each name one to three referents. However, the mentions seem to differ when indicating the influences of the female and male gender. In fact, regarding the mentions of the former, in 63% of the networks in which the ego is male, women are not mentioned. When men mention women, it is most common to refer to only one 29% of the time. On the other hand, 57% of the women mention at least one female peer. However, it is most frequent for women to mention only one female referent 39% of the time.

Concerning mentions of the male gender referents, 94% of men mentioned at least one referent of the same gender, whereas 89% of women mentioned also mentioned male referents. The analysis shows that such a trend is a reinforced tendency as more alters are mentioned only among men. In the case of women, it is most frequent for them to mention a referent of the male gender. Given the set of weighted mentions of each gender by male and female researchers, it is possible to construct the transition matrix shown in Table 7.

**Table 7:** Transition matrix of mentions by gender.

Criteria	Mentions to male referents	Mentions to female referents
Male (*n* = 281)	1.95/2.42 = 0.81	0.45/2.42 = 0.19
Female (*n* = 181)	1.60/2.40 = 0.67	0.78/2.40 = 0.33

**Figure 1:**
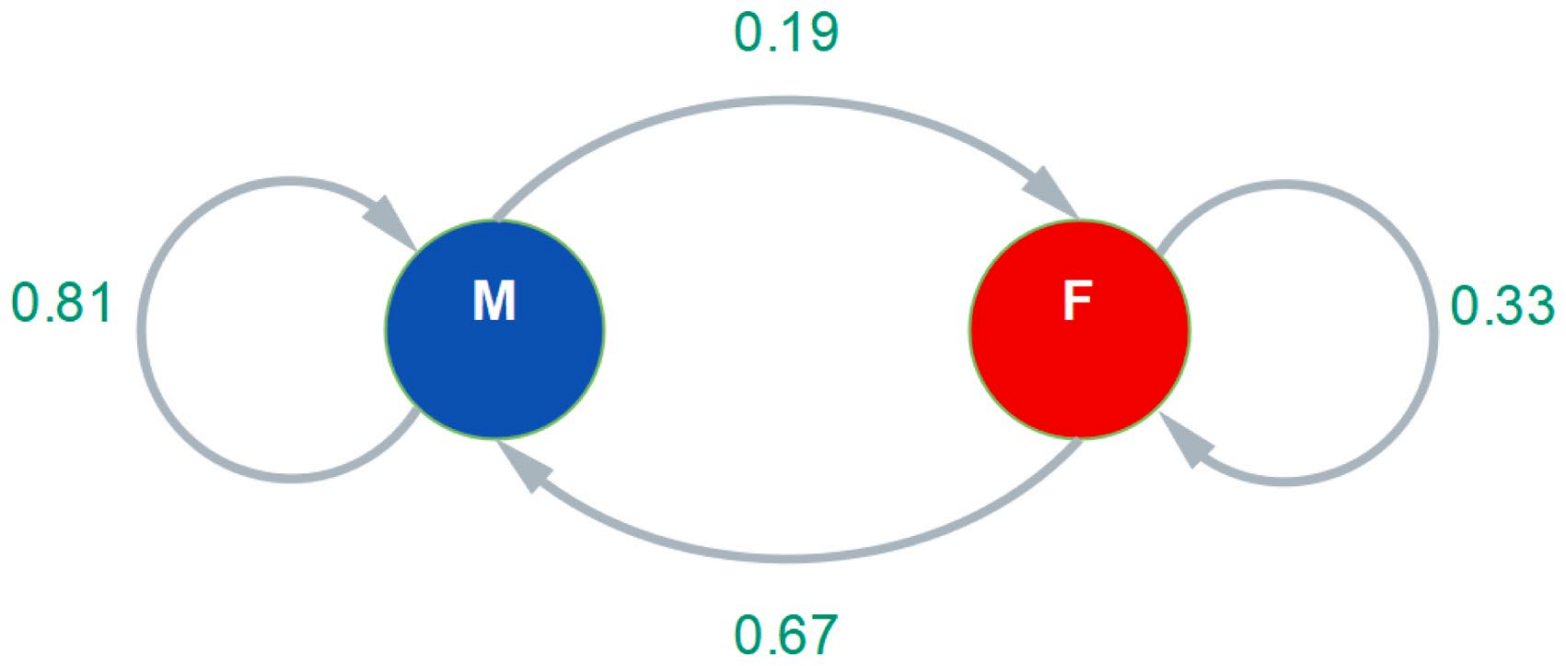
Graph with flow intensities for intragender and intergender mentions.

Figure 1 then represents these flow intensities in the transition matrix employing an irreducible directed graph, from which the authors derived the resulting long-term steady, which refers to 70% to men and 30% to women (See Eq. 1).

Equation 1 steady-state calculation


(1) $$\begin{array}{l} \pi ={\left[\begin{array}{l} 0.81\;0.19\\ 0.67\;0.33 \end{array}\right]}^{T}*\pi \\ \pi_{1}+\pi_{2}=1\\ \pi_{i}\geq 0 \end{array}$$

Indeterminate mentions are not a problem for both cases. So, performing homophily analysis if they represent the opposite gender can only generate results that tend to a value of 0 or +/−0.33, depending on whether the network has three mentions. Table 8 presents the Krackhardt and Stern index values distribution for men and women. In addition, the mean differs statistically between both groups at a confidence rate of 95%.

**Table 8:** Krackhardt and Stern index by gender.

Value	Total (*n* = 462)	Male (*n* = 281)	Female (*n* = 181)
−1 (Heterophily)	20%	6%	43%
−0.33	13%	6%	23%
0	6%	4%	10%
+0.33	19%	22%	14%
+1 (Homophily)	42%	62%	10%
Average	0.235	0.620	−0.363

Table 8 results clarify that the general tendency towards homophily is valid only for males. Female egocentric networks attenuate the average EI index value of the sample of 462 egocentric networks.

In the case of women, the tendency is toward heterophily; they tend to mention both women and men among people in Chile who have influenced their careers. However, it is noteworthy that 43% of women’s networks of influence are represented only by men. In 23% of the networks in which the ego is female, two-thirds are men. In 10% of the networks, there is a balance between men and women. Cases where women predominate within their networks, are only 24%, of which 14% are networks in which two-thirds are women and un 10% of all the referents are women.

On the other hand, in egocentric networks of men, the tendency is towards homophily; they usually tend to mention men among people in Chile who have influenced their careers. Subjects mentioned only men in 62% of their networks. Furthermore, in 22% of the networks, most members are men. Only 4% of networks are gender balanced. In 12% of the networks, the subjects of the study mainly mention female referents. Finally, in 6% of the subjects, two-thirds of their ego network comprises women, and the referents are all female in the same percentage.

The results of the homophily analysis, combined with the findings discussed above, suggest a potential negative feedback loop for women, however. If co-authorship is a powerful method for faculty members to signal their value to others, and if male faculty co-authors more with male faculty, it is more difficult for female faculty to show their value to their male colleagues. However, the indices calculated for each gender group reflect an overall tendency for men to collaborate more often with men and for women to collaborate more frequently with men. In other words, the literature shows that in the work context, men tend to have more homophilic networks than women.

## 4. Discussion

The egocentric networks analyzed here demonstrate a little-studied pattern of homophily, which responds to finding referents and influences in other people (McPherson et al., 2001). In this sense, we understand gender as a significant homophily factor in influencing scientific careers, and it may also play an essential role in other aspects of this type of activity. This homophily is related to the participation or awarding of projects, which, as expressed in this text, reproduce factors of inequality. A central result of this study indicates that men tend to find referents in other men, while women also do so about men. It is compelling that men tend to find referents in, mainly men. For example, in contrast to the case of gender homophily present in the study on hiring already reviewed (Edo et al., 2019). In that study, the authors observed that people of each gender tend to favor their congeners. In the case of the references in the scientific field in Chile, we see that this is not the case for women. Thus, women working in science show a preference for mentioning male referents.

Despite the extensive bibliography reporting gender gaps in science (Acker, 1990; Sanhueza-Díaz et al., 2020; Hajibabaei et al., 2022; Filandri et al., 2023) in the Chilean case, some of the social mechanisms underlying this phenomenon remain to be understood. In this sense, homophily as a social mechanism has been widely studied in social sciences, playing a significant role in different areas of human experience (Hedstrom and Swedberg, 1998), displayed clearly in egocentric networks. In the specific case of the Chilean scientific field, homophily of gender is evident; however, among female researchers, there is heterophily. Issues about the heterophily of gender in the Chilean scientific field potentially show two different phenomena. On the one hand, it could be the case that the reproduction of stereotypes is expressed in scientific activity to the male gender, thus favoring the recruitment and selection of people consistent with these stereotypes.

On the other hand, this tendency can hinder women’s attempts to pursue scientific careers by understanding the field as dominated by androcentric logic and male referents. In the Chilean case, some evidence shows that it is at primary school that women build their interests in science. In those early years, the female science and math teacher figure is paramount for girls’ preferences (Blázquez et al., 2009). This evidence is aligned with some recent international results on the study of network collaboration in the long term, where gender inequalities seem to be related to the lack of women in leadership positions (Bellotti et al., 2022). Furthermore, this phenomenon also echoes results in the postdoctoral hiring of STEMM fields, where hiring disparities correlate with between-group differences in applicants’ network connections, referrer prestige, and academic human capital (Shauman and Huynh, 2023), and authors demonstrated that intergender collaboration increases male and decrease female scholar research performance (Shen et al., 2022). All in all, reflection on women in STEM has highlighted both phenomena as intervening in gender gaps in these areas of knowledge (UNESCO, 2017a,b; Thébaud and Charles, 2018).

Any reader of this study must consider some specific cautions when interpreting the results of a study such as the one presented here. This caution relates to the scarcity of additional information on the egos; their alters, and the relationships between the two. Such scarce information means that readers of this article should interpret any results presented here as a tendency toward homophily regarding gender. This study lacks certain information that would be relevant to comprehensively studying patterns of homophily, as the present study’s authors do not construct the instrument of input for this purpose. For example, the degree of closeness between the ego and alters, the frequency of contact or interaction, whether they have worked on a project together, and the individual characteristics of the alter, among others. On the other hand, this is not a statistically representative sample of the scientific community in Chile.

Finally, some literature has shown inconsistent performances in homophily and heterophily graphs (Maurya et al., 2022). For this reason, readers need to understand this study’s results as a description of the networks of the 594 participants. Likewise, referents are not strictly scientific, given that the statement did not explicitly refer to them. However, most of the answers were oriented this way, although other generic answers, such as “my mother” or “my sister,” were presented.

After considering the already presented warns, results highlight the value of homophily as a factor in the reproduction of asymmetries within society, insofar as the authors have not seen that people of one gender identify mechanically with people of the same gender. However, this occurs for men and not for women, with possible consequences as those described above. In this way, not only is homophily relevant, but also how it interacts with heterophily. In addition, for the Chilean case, despite the extensive bibliography reporting gender gaps in science (Acker, 1990; Sanhueza-Díaz et al., 2020), some of the social mechanisms underlying this phenomenon remain to be understood. However, and as a precautionary measure, heterophily, when related to the father figure, maybe indicate a referent of an aspirational nature. In this sense, in the present study, the role has not been analyzed in this aspirational character. The data are mostly given by name and alias, and there is a limitation in the study since it is not possible to deduce what is the relationship that ego has with each of these alters.

## 5. Conclusion

This study provides a first approach to the problem of gender influence and referents in science in Chile. The contribution of this study is the characterization of the network of male and female scientists and how they relate to each other based on the open mention of their referents in their scientific careers. In doing so, the text reveals a constitutive aspect of the network of scientists studied. In this sense, the data discussed in the results section show clear trends in how the people who participate in this field evaluate the influence of other people in terms of gender homophily. We conclude that it is very likely that this heterophilic and homophilic mechanism, in which scientists related to their referents, plays a vital role in reproducing the inequalities and asymmetries constitutive of the Chilean scientific field.

Although the study could have a broader scope if it had a more comprehensive sample, the work is a contribution that demonstrates how references are produced and reproduced, a significant aspect of the barriers that women encounter in their scientific careers. On the other hand, the authors based the present study not on collaborations between male and female researchers but on spontaneous mentions. Although this presents some disadvantages in terms of specific people not answering or mentioning people not related to science, the experience provided consistent information that does not have the levels of error presented, for example, by research based on collaborations studied based on bibliometric studies (Halevi, 2019).

Likewise, there is no doubt that the problem charted here is dynamic. Scientific communities have recently been substantively incorporating Women into the scientific field in a context of cultural change that highlights the role of women in all areas. Thus, monitoring the change in results over time will be necessary. Such continuous monitoring is crucial because the more the scientific sector incorporates women into scientific activity, the more women will be able to position themselves as referents for other women and men. This way, it will be possible to provide a longitudinal analysis of the problem studied in this text. In this sense, relieving a practical point of view, this study contributes to greater social sustainability, being able to be used as an argumentative basis for creating equality programs in science policy and education that achieve more equalitarian opportunities for men and women (SDG 5).

A policy of gender equity in science, such as the one that the Chilean Ministry of Science, Knowledge, Technology, and Innovation has been developing (Biblioteca Nacional del Congreso, 2020), should take these aspects into account, making visible the women who stand out in the scientific field and encouraging female participation and leadership within research teams. Such developments will contribute to forming an inclusive scientific field that takes advantage of and enhances the talents of the entire population.

## Data availability statement

The original contributions presented in the study are included in the article/Supplementary material, further inquiries can be directed to the corresponding author.

## Ethics statement

The studies involving humans were approved by Comité de Ética Institucional Universidad de Santiago de Chile. The studies were conducted in accordance with the local legislation and institutional requirements. The participants provided their written informed consent to participate in this study. Written informed consent was obtained from the individual(s) for the publication of any potentially identifiable images or data included in this article.

## Author contributions

JE-C: conceptualization, formal analysis, and project administration. AV-M and JE-C: methodology and validation. LL-J and AV-M: software. AV-M: data curation. JE-C and LC-S: writing – original draft preparation. JE-C, LC-S, and AV-M: writing – review, and editing and funding acquisition for publishing fees. All authors contributed to the article and approved the submitted version.

## Funding

The publication was funded by the Chilean ANID project: Fondecyt 1190543: “Doing Laboratory Studies in Chile: Re-engaging Science in the Making.”

## Conflict of interest

The authors declare that the research was conducted in the absence of any commercial or financial relationships that could be construed as a potential conflict of interest.

## Publisher’s note

All claims expressed in this article are solely those of the authors and do not necessarily represent those of their affiliated organizations, or those of the publisher, the editors and the reviewers. Any product that may be evaluated in this article, or claim that may be made by its manufacturer, is not guaranteed or endorsed by the publisher.
